# Treatment of Pyoderma Gangrenosum in Pediatric Inflammatory Bowel Disease

**DOI:** 10.1097/PG9.0000000000000008

**Published:** 2020-08-21

**Authors:** Katherine Vaidy, Rebecca Winderman, Simon S. Rabinowitz, Steven M. Schwarz

**Affiliations:** From the Division of Gastroenterology, Hepatology and Nutrition, Children’s Hospital at Downstate, SUNY-Downstate Health Sciences University, Brooklyn, NY.

**Keywords:** adalimumab, corticosteroids, immunomodulators, infliximab, inflammatory bowel disease, tumor necrosis factor-α

## Abstract

Pyoderma gangrenosum (PG) is a rare, necrotizing dermatologic condition associated with neoplastic and immune dysregulatory states, including adult and pediatric inflammatory bowel disease (IBD). Over the last decade, the elucidation of inflammatory mediators in PG has led to a plethora of localized and systemic corticosteroid sparing therapies including antibiotics, antiinflammatory, and immunomodulatory agents. Herein, we describe the case of a 17-year-old female with ulcerative colitis in clinical remission, who presented with a long-standing, large, deep, and painful lower extremity PG lesion. Following failed attempts both at local and at systemic therapies, her PG was successfully treated with the tumor necrosis factor-alpha (TNF-α) monoclonal antibody adalimumab, and the lesion remains in remission after four years of subcutaneous anti-TNF therapy. This case serves as the basis for our presenting a review of the pathogenesis, diagnostic criteria, differential diagnosis, therapies and treatment outcomes for pediatric IBD-associated PG. Our experience adds to earlier reports suggesting anti-TNF-α biologic therapy is most likely to achieve long-term resolution of IBD-associated PG in children and adolescents with severe lesions or who failed other treatments.

## CASE REPORT

**FIGURE 1.**
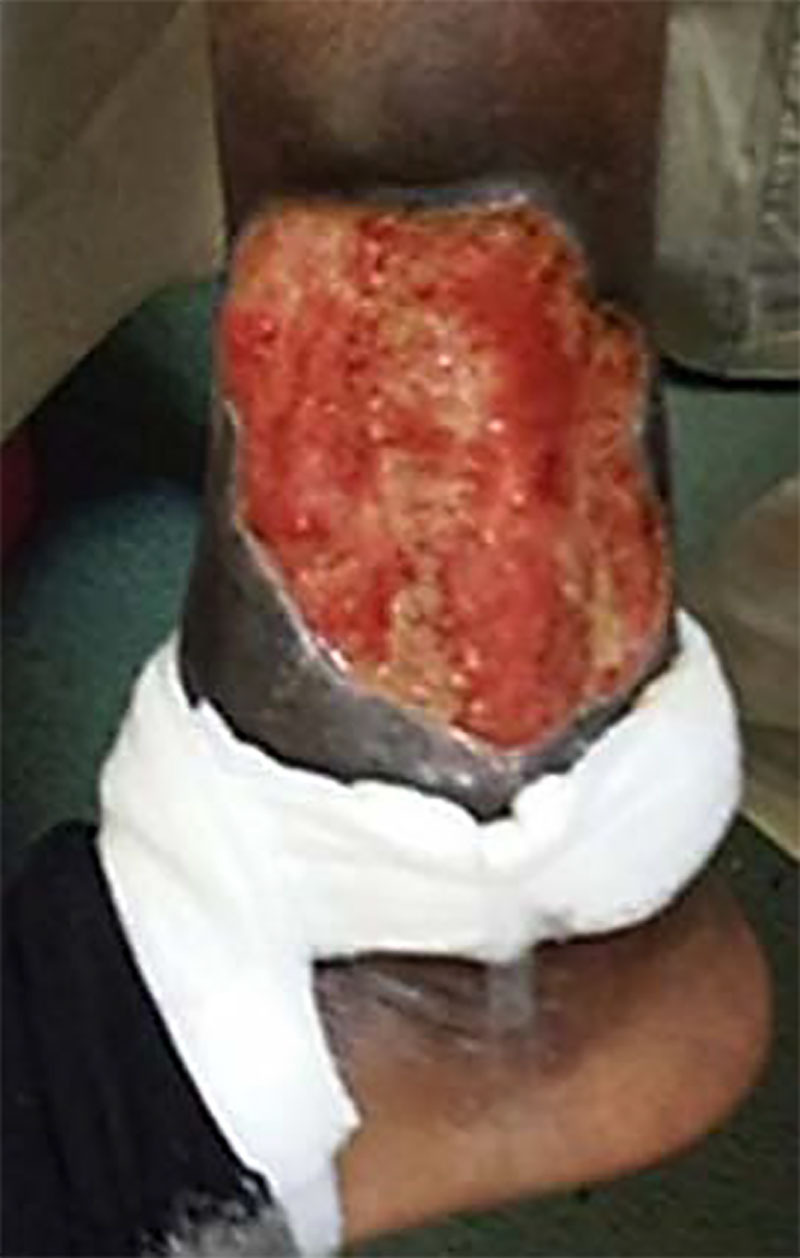
Pyoderma gangrenosum lesion at presentation.

A 17-year-old female, a recent émigré from Jamaica, was referred to the Pediatric Gastroenterology service at the Children’s Hospital, SUNY-Downstate Medical Center, for management of ulcerative colitis (UC) in clinical remission. Her treatment comprised only oral prednisone 5 mg daily at the time of consultation. A diagnosis of UC had been made at age 10 years, and, at 13 years of age, she was diagnosed with a left lower extremity “venous stasis ulcer,” which had progressed despite multiple courses of therapy with antibiotics and topical corticosteroids. At the time of referral, she denied symptoms of weight loss, diarrhea, bleeding and abdominal pain, and her Pediatric Ulcerative Colitis Activity Index (PUCAI) score was 0. Her chief complaint was the painful leg “ulcer.” Physical examination demonstrated a well-developed, well-nourished young woman, Tanner Stage V. The remainder of the examination was remarkable only for a deep, left lower leg lateral and pretibial ulcerating lesion measuring approximately 15 × 7 cm (Fig. 1). The ulceration was characterized by a violaceous border, purulent exudate, and areas of necrosis, with what appeared to be exposed connective tissue. These physical findings were consistent with severe Pyoderma gangrenosum (PG) in the setting of UC, and the diagnosis of PG was confirmed histopathologically.

Initial laboratory studies included a hemoglobin of 12.0 gm/dL, serum albumin of 4.4 g/dL (normal 3.5–5.7 g/dL) and marked elevations both of the erythrocyte sedimentation rate (>130 mm/h) and of C-reactive protein (>40 mg/L). Serologic evaluation demonstrated elevated perinuclear antineutrophil cytoplasmic antibody (p-ANCA) and negative anti-*Saccharomyces cerevisiae* antibody (ASCA) titers. Colonoscopy showed some loss of the normal vascular pattern but appeared otherwise unremarkable (Mayo endoscopic score 1). Histopathology showed mild acute and chronic inflammation with occasional cryptitis in the rectosigmoid and descending colon. Ileal biopsies were normal. These endoscopic findings of mild colitis were consistent with her prior UC diagnosis, and treatment was started with an oral 5-aminosalicylic acid (5-ASA) derivative.

**FIGURE 2.**
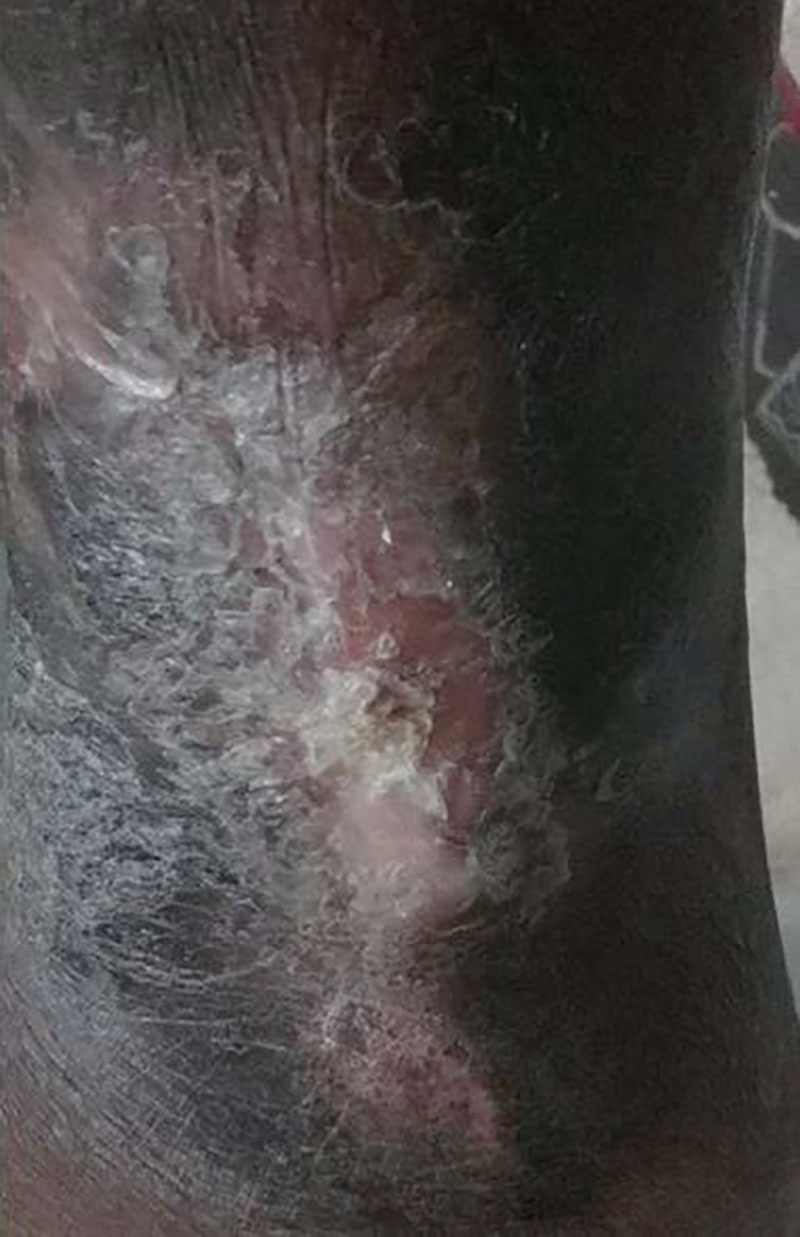
Pyoderma gangrenosum after 6 months of therapy with adalimumab. The healed lesion demonstrates a central area of hypotrophic epithelialization and peripheral cribriform scarring.

Over a 1-year period, the PG lesion was refractory to multiple therapies, including oral and intralesional corticosteroids, topical and oral tacrolimus, topical dapsone, oral antibiotics (including metronidazole and doxycycline), and intralesional interleukin-10 (IL-10). Subsequently, the family sought consultation at a regional burn center, where autologous skin grafting was performed and was unsuccessful. Her colitis remained in clinical remission, with a persistent PUCAI score of 0. Subcutaneous adalimumab (ADA) monotherapy was then initiated, using a standard induction and maintenance protocol. Significant improvement in the PG ulcer was observed within 1 month of commencing treatment, with almost complete healing after 6 months (Fig. 2). Adalimumab therapy was continued at 40 mg subcutaneously every 2 weeks. Both UC and PG have remained in clinical remission after 4 years, and no new PG lesions have appeared.

## PYODERMA GANGRENOSUM IN INFLAMMATORY BOWEL DISEASE

### Method of Review

In collecting reports of PG diagnosis and management in pediatric inflammatory bowel disease (IBD), data were accessed from MedlinePlus and the National Institutes of Health US National Library of Medicine, to review IBD-associated PG treatment in children and adolescents, reported in English language articles published between 2002 and 2018. Inclusion criteria comprised: (1) age ≤19 years at diagnosis of histopathologically confirmed PG; (2) PG associated with a confirmed case of IBD; (3) indication of specific PG treatments and outcomes. We reviewed prospective, randomized controlled trials, population-based and observational studies, metaanalyses and systematic reviews appearing in peer reviewed journals and cited at https://www.ncbi.nlm.nih.gov/pubmed/. This search included pediatric patients reported in adult case series. Thirty-one references were identified, of which 9 were eliminated as lacking a discussion of specific clinical cases. Ten references^1-10^ were excluded because they did not satisfy all of the above inclusion criteria. Twelve published studies met all search criteria and described a total of 19 patients (13 females, 6 males). These reported cases included 13 patients with Crohn’s disease (CD) and 6 with UC.

### Clinical Presentation and Diagnosis

Pyoderma gangrenosum is a neutrophilic dermatosis that may develop *de novo* or, more commonly, in the clinical setting of an underlying inflammatory or neoplastic disease. The worldwide incidence of PG is estimated to be 3–10 cases per 1,000,000 population per year^11^ and 4% are in children.^12^ In adults, approximately 60% of reported PG cases occur in association with a systemic disorder, including rheumatoid arthritis, autoimmune hepatitis, and malignancy. Up to 50% are associated with IBD^11-13^; however, <5% of patients with IBD develop PG.^11,13^ In IBD patients, PG is diagnosed most frequently in association with UC, but it is also reported in CD.^12,13^ It is more common in female patients and in those with active colitis.^12^ The lesion’s characteristics include a deep, extremely painful, papulopustular, necrotic cutaneous ulcer with an undermined and violaceous border that frequently heals with cribiform scarring.^12,13^ The occurrence and severity of PG lesions may not directly parallel IBD clinical activity.^12,14,15^ In adults, PG most commonly occurs on the lower extremities, whereas in children, it frequently appears on the head and in the genital, peristomal, and perianal regions.^16–18^ Lesions may develop acutely or over the course of months to years and may recur after apparently successful treatment.^13,14^

A diagnosis of PG is based on clinical features and the exclusion of other causes of cutaneous ulcerations including infection, vascular disease, other neutrophilic dermatoses, and malignancy,^13,14,16,18–20^ as listed in Table 1. Histologically, the PG lesion is composed of a dense neutrophilic dermal infiltrate, with marked tissue necrosis at the base of the ulcer.^21^ Up to 30% of cases mimic the cutaneous findings seen in neoplastic and in other inflammatory disorders .^13,14,16^ Diagnostic criteria for PG were proposed in 2018, through a consensus of selected international experts, using Delphi methodology, and these are presented in Table 2.^20^ A confirmed case of PG requires identification of the 1 major criterion and at least 4 of 8 minor clinical, histologic, and treatment criteria.

**Table 1.**
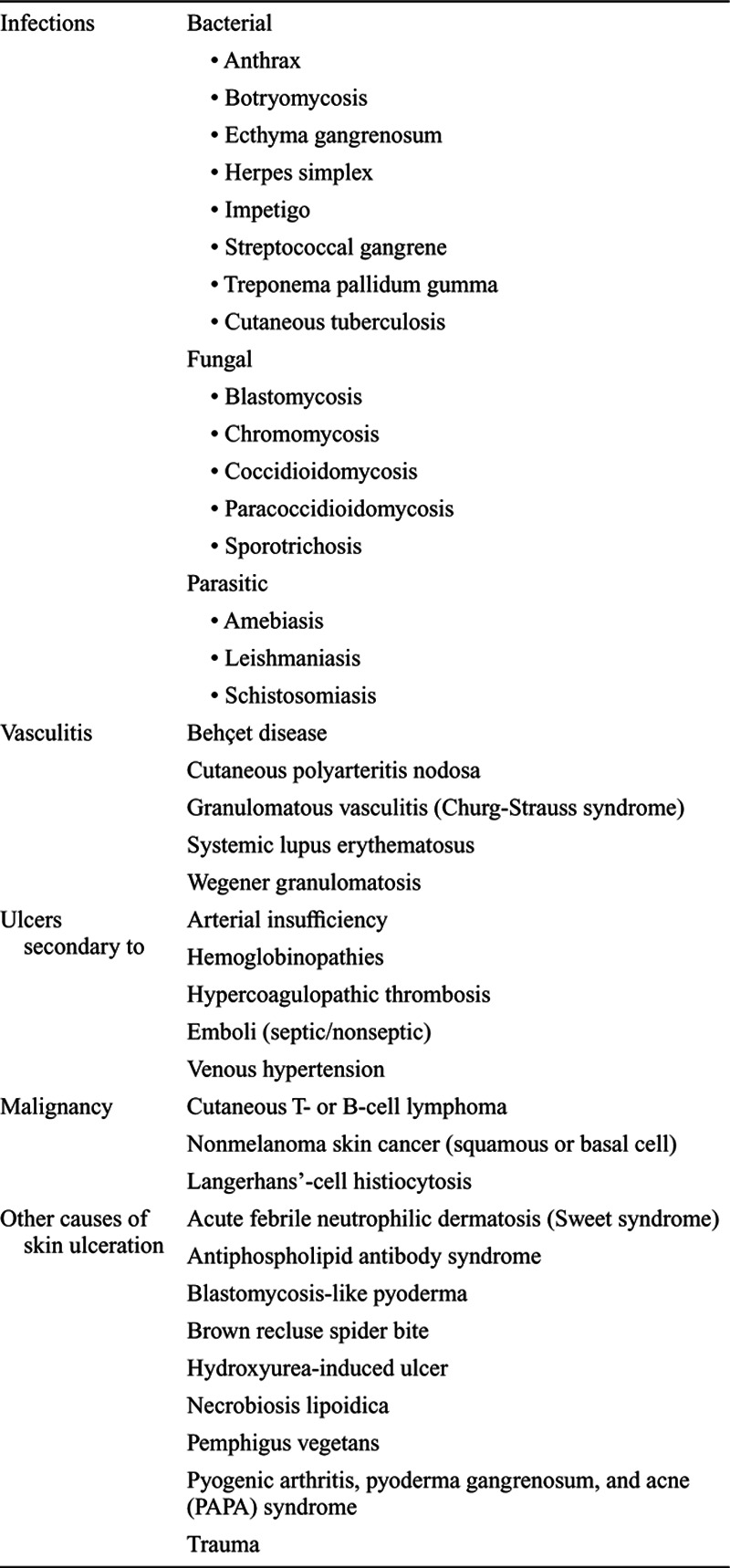
Differential Diagnosis of Pyoderma Gangrenosum•

**Table 2.**
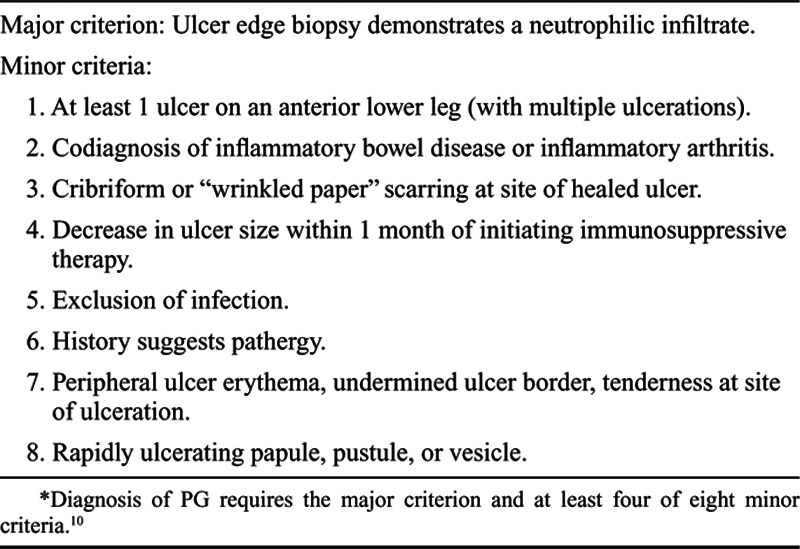
Proposed Diagnostic Criteria for Pyoderma Gangrenosum (PG)*

### Pathogenesis/Genetics

**FIGURE 3.**
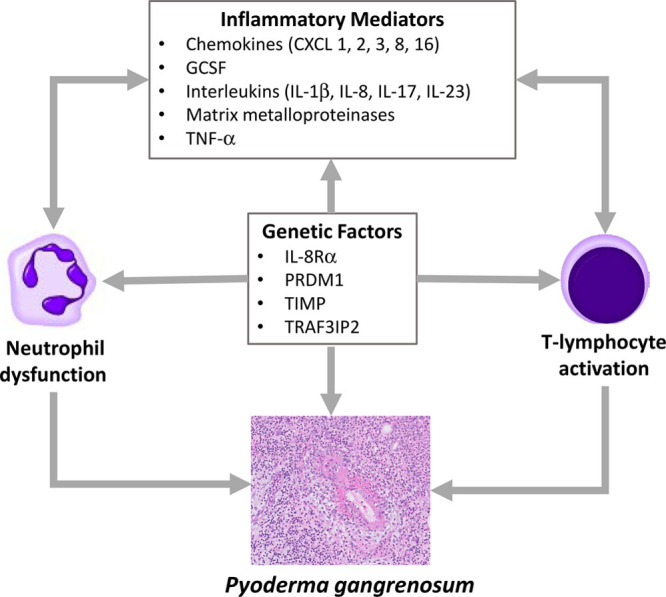
Proposed interrelationships among proinflammatory and genetic factors involved in the pathogenesis of IBD-associated pyoderma gangrenosum. CXCL, C-X-C motif chemokine ligand; GCSF, granulocyte-colony stimulating factor; IL-8Rα, interleukin-8 receptor alpha; PRDM1, PR domain-containing protein 1; TIMP, tissue inhibitor of metalloproteinase; TNF-α, tumor necrosis factor-alpha; TRAF3IP2, tumor necrosis factor receptor-associated factor 3 interacting protein 2.

The Th1 type of inflammatory process noted in PG is characterized by a complex interplay of cytokines, neutrophils, and T lymphocytes.^13,22–24^ Dysfunction both of the innate and of the adaptive immune systems causes upregulation of granulocyte-colony stimulating factor (GCSF) and of diverse cytokine/chemokine/interleukin (IL) moieties, including Tumor necrosis factor-alpha (TNF-α). Pathergy, a condition in which an environmental trigger, such as minor trauma, results in an exaggerated immune response, represents one of the PG minor criteria.^13,23^ This cascade of immune-mediated events may explain why PG has commonly been associated with other autoimmune diseases.^13,15,23,25^ Genome wide studies, examining the association of IBD with PG, have identified potential etiopathogenic factors, including regulatory sites for IL-8Rα (IL-8 receptor α, a mediator of neutrophil migration), PR domain-containing protein 1 (associated with the pathogenesis of autoimmune disease), and tissue inhibitor of metalloproteinase.^23^ Possible interrelationships among these varied inflammatory mediators and genetic determinants are presented in Figure 3.

## TREATMENT OF PYODERMA GANGRENOSUM

Treatment of PG is targeted at ameliorating the inflammatory process causing the ulcerative lesion. Several factors may influence therapeutic choices, including the number and size of PG lesions, the clinical course of disease (indolent vs more rapidly progressive), the presence of associated illnesses, and other comorbid conditions. Published reports in adult PG populations have shown relatively small and slowly progressive PG lesions often may be successfully managed with local therapy, including the use of topical corticosteroids, calcineurin inhibitors, and antibiotics.^26^ Systemic treatment has generally been required for larger and more aggressive cases. Because of the paucity of well-controlled studies, particularly in pediatric subjects, definitive PG treatment guidelines have not been established. As described above, Table 3 lists the 12 published reports of pediatric IBD-associated PG that include patient characteristics, histopathologically confirmed IBD and IBD-associated PG, cutaneous location of PG lesion(s), as well as initial and second-line PG therapies. Most cases of severe PG did not respond either to local wound care or to intralesional injections, but multiple pediatric cases of IBD-associated PG achieved a complete clinical response with an anti-TNF-α biologic agent.

**Table 3.**
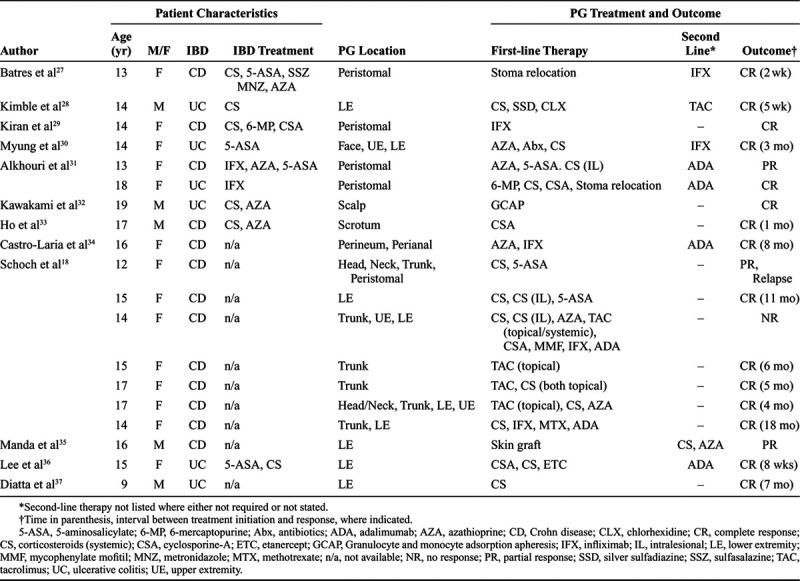
Pyoderma Gangrenosum (PG) Treatments and Outcomes in 19 Pediatric Patients With Inflammatory Bowel Disease (IBD)

### Topical/intralesional Agents

In the management of PG, topical/intralesional agents, including calcineurin inhibitors and corticosteroids (most commonly triamcinolone) have been used in combination with a systemic drug.^12,38^ In the present review, 3 patients receiving topical tacrolimus responded completely when combined with systemic corticosteroids; and, one patient achieved a complete PG remission with topical tacrolimus monotherapy.^28,38^ Local therapy of pediatric PG with topical or with intralesional agents may be expected to ameliorate smaller and more indolent lesions, as indicated in prior reports of adult IBD-associated PG.^26^

### Corticosteroids

High-dose, systemic corticosteroids are effective in halting the progression of PG and, therefore, have been recommended as first-line treatment.^13,14,39^ However, frequent PG exacerbations/recurrences following treatment discontinuation may compromise steroid utility in long-term PG management, particularly in pediatric patients.^39^ In the present review, 11 patients received systemic corticosteroids for management of PG, with only 1 patient experiencing a complete response with monotherapy.^18,28,30,31,36^

### Calcineurin Inhibitors

Cyclosporine-A (CSA) has been recommended as a first-line treatment for IBD-associated PG, because of its role in inhibiting T-cell activation, inflammatory cell proliferation, and proinflammatory cytokine production.^13,14,39^ In a randomized controlled trial comparing systemic corticosteroids to CSA for treating PG in adults, both treatments were associated with a clinical response rate of approximately 50%.^39^ In the present review, CSA monotherapy was successful in achieving a complete response in only 1 of 4 patients with PG.^28,31,33,36^ Tacrolimus, a potent calcineurin inhibitor, was administered orally to 2 reported pediatric patients, either alone or in combination with other immunomodulators. Of these, 1 patient achieved a complete response with systemic tacrolimus monotherapy.^18,28^

### Thiopurines and Methotrexate

The thiopurine drugs 6-mercaptopurine and azathioprine have commonly been used in the treatment of IBD-associated PG. However, despite their immunomodulatory effects, thiopurines alone have generally not been shown to be effective in treating IBD-associated PG, either in adults or in the 5 reported pediatric patients.^15^ The 1 pediatric case that showed clinical improvement also received systemic corticosteroids.^18^ Interestingly, 4 pediatric patients have been described who developed PG while undergoing thiopurine treatment for IBD, a complication previously seen in adults.^27,29,32,36^ Methotrexate monotherapy has not been widely used in the management of PG in the setting of IBD. Table 1 includes the report of a pediatric IBD patient who received methotrexate in combination with systemic corticosteroids and with anti-TNF-α biologic agents. The PG reached clinical resolution after 18 months of this combination therapy.^18^

### Anti-TNF-α Biologics

TNF-α is a potent inflammatory mediator found in PG lesions.^21,23-25^ This cell signaling protein upregulates proinflammatory factors, including IL-1β and IL-8,^40^ that act as mediators of inflammation in IBD and in PG.^15,17–20,41^ TNF-α also may stimulate cutaneous recruitment of inflammatory cells via enhanced vasculature expression of adhesion molecules, including the metalloproteinases MMP-9 and MMP-10.^21,24,25^ The use of anti-TNF-α biologics as first-line therapy for IBD-associated PG has been recommended, as PG severity may be independent of IBD clinical activity.^15,42–46^ Clinical reports also have demonstrated the efficacy of infliximab and adalimumab therapy of PG in other immune-mediated disorders,^19,30,33^ The nine reported pediatric and adolescent patients who received an anti-TNF-α biologic agent for IBD-associated PG are listed in Table 3. Eight achieved complete PG resolution with monotherapy, including those who failed other therapeutic regimens.^18,27,29–31,34,36^ These results are consistent with findings from larger studies in adults. In 1 review, all 13 adult patients with medically refractory PG responded to standard induction dosing with infliximab.^47^ The only randomized control trial of PG treatment to date included 29 adult subjects and reported a 69% PG response rate to infliximab.^48^ In the largest systematic review in the literature, anti-TNF-α PG therapy was associated with a 92% response rate among 40 adult IBD patients.^15^

### Other Therapies

In the present review, 6 patients received a 5-ASA drug (1 as monotherapy), without clinical improvement.^18,27,30,31,36^ Granulocyte and monocyte adsorption apheresis (GCAP) involves extracorporeal adsorption and removal of active granulocytes and monocytes from the circulation.^49,50^ Several small case series in adults and in 1 pediatric patient have demonstrated some efficacy in treating IBD-associated PG with GCAP.^32,49,50^ Intravenous immunoglobulin (IVIG) has been shown to result in a complete or partial clinical response in up to 87.8% of adults with refractory PG. However, only 1 reported pediatric patient with idiopathic (non-IBD related) PG has received such treatment.^38,51^ As in the case presented here, skin grafting has proven ineffective in both adults and pediatric patients with PG, and it may lead to worsening of PG as the consequence of tissue injury associated with the grafting procedure.^35^ Ustekinumab, a human monoclonal antibody targeting the p40 subunit of IL-12 and IL-23 has been used successfully in several adult cases of PG.^52^

## CONCLUSIONS

Pediatric PG is a rare extraintestinal manifestation of IBD that carries significant physical and psychologic morbidity and is often refractory to multiple treatments. Previously, management of pediatric PG has emphasized local wound care and the use of immunomodulatory drugs, including corticosteroids and calcineurin inhibitors,^18,28,33,35,37,38^ as well as systemic and topical antiinfective agents.^18,28,35,37,38^ However, earlier reports demonstrated little consistency in treatment protocols or in therapeutic efficacy.^18,28,33,35,37,38^ More recently, elucidation of the inflammatory cascade involved in the etiology and progression of PG has established a prominent role for TNF-α dysregulation; this observation serves as the basis for the use of anti-TNF-α biologics as first-line PG treatment. Although milder, indolent PG lesions may respond to local therapy, the case detailed herein, along with other published reports, have confirmed the effectiveness of TNF-α monoclonal antibodies in more extensive pediatric IBD-associated PG. Ideally, an organized, multicenter study could evaluate therapeutic alternatives in a randomized, controlled trial of systemic PG treatments in pediatric IBD subjects. However, until such an investigation can be conducted, currently available published data support using an anti-TNF-α biologic agent as first-line therapy for severe PG therapy in pediatric IBD, as well as for those cases that have not responded to local therapies.

## References

[1] Nozawa T Hara R Kinoshita J. Infliximab for a girl with refractory pyoderma gangrenosum. Nihon Rinsho Meneki Gakkai Kaishi. 2008; 31: 454–459 19122376 10.2177/jsci.31.454

[2] Hubbard VG Friedmann AC Goldsmith P. Systemic pyoderma gangrenosum responding to infliximab and adalimumab. Br J Dermatol. 2005; 152: 1059–061 15888172 10.1111/j.1365-2133.2005.06467.x

[3] Androutsakos T Stamopoulos P Aroni K. A case report of successful treatment of pyoderma gangrenosum in a patient with autoimmune hepatitis, and review of the literature. BMC Gastroenterol. 2015; 15: 49 26502871 10.1186/s12876-015-0376-1 PMC4624371

[4] Franco S Foresta G Pulvirenti E Giannone G. Ulcerative colitis complicated by atypical peristomal pyoderma gangrenosum. Inflamm Bowel Dis. 2011; 17: e111–e112 21630389 10.1002/ibd.21784

[5] Ermis F Ozdil S Akyüz F Pinarbasi B Mungan Z. pyoderma gangrenosum treated with infliximab in inactive ulcerative colitis. Inflamm Bowel Dis. 2008; 14: 1611–613 18401859 10.1002/ibd.20481

[6] Ben Chaabane N Hellara O Ben Mansour W. Auricular pyoderma gangrenosum associated with Crohn’s’s disease. Tunis Med. 2012; 90: 414–415 22585655

[7] Sinagra E Orlando A Renna S Maida M Cottone M. Multifocal pyoderma gangrenosum resistant to infliximab in active ulcerative colitis: don’t forget the role of cyclosporin. Inflamm Bowel Dis. 2012; 18: e1594–e1595 22038892 10.1002/ibd.21915

[8] Handler MZ Hamilton H Aires D. Treatment of peristomal pyoderma gangrenosum with topical crushed dapsone. J Drugs Dermatol. 2011; 10: 1059–1061 22052278

[9] Hurabielle C Schneider P Baudry C Bagot M Allez M Viguier M. Certolizumab pegol—a new therapeutic option for refractory disseminated pyoderma gangrenosum associated with Crohn’s’ disease. J Dermatolog Treat. 2016; 27: 67–69 25909366 10.3109/09546634.2015.1034075

[10] Teich N Klugmann T. Rapid improvement of refractory pyoderma gangrenosum with infliximab gel in a patient with ulcerative colitis. J Crohn’s Colitis. 2014; 8: 85–86 23810275 10.1016/j.crohns.2013.06.003

[11] Cozzani E Gasparini G Parodi A. Pyoderma gangrenosum: a systematic review. G Ital Dermatol Venereol. 2014; 149: 587–600 25213386

[12] Rosmaninho A Carvalho S Teixeira V. Pyoderma gangrenosum: a mini-review. Eur Med J. 2015; 3: 79–86

[13] Weizman A Huang B Targan S. Pyoderma gangrenosum among patients with inflammatory bowel disease: a descriptive cohort study J Cutan Med Surg. 2014; 18: 361 PMC5089702 25277124

[14] Ruocco E Sangiuliano S Gravina AG. Pyoderma gangrenosum: an updated review. J Eur Acad Dermatol Venereol. 2009; 23: 1008–1017 19470075 10.1111/j.1468-3083.2009.03199.x

[15] Agarwal A Andrews JM. Systematic review: IBD-associated pyoderma gangrenosum in the biologic era, the response to therapy. Aliment Pharmacol Ther. 2013; 38: 563–572 23914999 10.1111/apt.12431

[16] Su WPD Davis MD Weenig RH. Pyoderma gangrenosum: clinicopathologic correlation and proposed diagnostic criteria. Internat J Dermatol. 2004; 43: 790–800 10.1111/j.1365-4632.2004.02128.x 15533059

[17] Brooklyn T Dunnill G Probert C. Diagnosis and treatment of pyoderma gangrenosum. Br Med J. 2006; 333: 181–184 16858047 10.1136/bmj.333.7560.181 PMC1513476

[18] Schoch JJ Tolkachjov SN Cappel JA. Pediatric pyoderma gangrenosum: a retrospective review of clinical features, etiologic associations, and treatment. Pediatr Dermatol. 2017; 34: 39–45 27699861 10.1111/pde.12990

[19] Moschella SL Davis MD. Bolognia JL Jorizzo JL Rapini RP. Neutrophilic Dermatoses. Dermatol. 2008. 2nd ed, Barcelona: Mosby Elsevier

[20] Maverakis E Ma C Shinkai K. Diagnostic criteria of ulcerative pyoderma gangrenosum: a delphi consensus of international experts. J Amer Med Assoc Dermatol. 2018; 154: 461–466 10.1001/jamadermatol.2017.5980 29450466

[21] Marzano AV Cugno M Trevisan V. Role of inflammatory cells, cytokines and matrix metalloproteinases in neutrophil-mediated skin diseases. Clin Exp Immunol. 2010; 162: 100–107 20636397 10.1111/j.1365-2249.2010.04201.x PMC2990935

[22] Brooklyn TN Williams AM Dunnill MG Probert CS. T-cell receptor repertoire in pyoderma gangrenosum: evidence for clonal expansions and trafficking. Br J Dermatol. 2007; 157: 960–966 17935516 10.1111/j.1365-2133.2007.08211.x

[23] Braswell SF Kostopoulos TC Ortega-Loayza AG. Pathophysiology of pyoderma gangrenosum (PG): an updated review. J Amer Acad Dermatol. 2015; 73: 691–698 26253362 10.1016/j.jaad.2015.06.021

[24] Marzano AV Fanoni D Antiga E. Expression of cytokines, chemokines and other effector molecules in two prototypic autoinflammatory skin diseases, pyoderma gangrenosum and Sweet’s syndrome. Clin Exp Immunol. 2014; 178: 48–56 10.1111/cei.12394 PMC4360193 24903614

[25] Wang EA Steel A Luxardi G. Classic ulcerative pyoderma gangrenosum is a T cell-mediated disease targeting follicular adnexal structures: a hypothesis based on molecular and clinicopathologic studies. Front Immunol. 2018; 15: 1980 10.3389/fimmu.2017.01980 PMC5775228 29379508

[26] Thomas KS Ormerod AD Craig FE. Clinical outcomes and response of patients applying topical therapy for pyoderma gangrenosum: a prospective cohort study. J Am Acad Dermatol. 2016; 75: 940–949 27502313 10.1016/j.jaad.2016.06.016

[27] Batres L Mamula P Baldassano RM. Resolution of severe peristomal pyoderma gangrenosum with infliximab in a child with Crohn’s disease. J Pediatr Gastroenterol Nutr. 2002; 34: 558–560 12050585 10.1097/00005176-200205000-00016

[28] Kimble RM Tickler AK Nicholls VS Cleghorn G. Successful topical tacrolimus (FK506) therapy in a child with pyoderma gangrenosum. J Pediatr Gastroenterol Nutr. 2002; 34: 555–557 12050584 10.1097/00005176-200205000-00015

[29] Kiran RP O’Brien-Ermlich B Fazio VW Delaney CP. Management of peristomal pyoderma gangrenosum. Dis Colon Rectum. 2005; 48: 1397–403 15868233 10.1007/s10350-004-0944-x

[30] Myung IM Juillerat P Christen-Zach S. Infliximab for the treatment of disseminated pyoderma gangrenosum associated with ulcerative colitis. Case report and literature review. Dermatol. 2007; 215: 245–251 10.1159/000106584 17823524

[31] Alkhouri N Hupertz V Mahajan L. Adalimumab treatment for peristomal pyoderma gangrenosum associated with Crohn’s’s disease. Inflamm Bowel Dis. 2009; 15: 803–806 18942748 10.1002/ibd.20748

[32] Kawakami T Yamazaki M Soma Y. Reduction of interleukin-6, interleukin-8, and anti-phosphatidylserine-prothrombin complex antibody by granulocyte and monocyte adsorption apheresis in a patient with pyoderma gangrenosum and ulcerative colitis. Am J Gastroenterol. 2009; 104: 2363–64 19727102 10.1038/ajg.2009.271

[33] Ho SA Tan WP Tan AW. Scrotal pyoderma gangrenosum associated with Crohn’s disease Singapore Med J. 2009; 50: e397–e400 20087538

[34] Castro-Laria L Arguelles-Arias F Arguelles-Martin F Herrerias-Gutierrez JM. Perineal pyoderma gangrenosum in a girl treated with adalimumab after infliximab failure. Rev Esp Enferm Dig. 2011; 103: 439–441 21867361 10.4321/s1130-01082011000800015

[35] Manda G Finch P Mponda K. Pyoderma gangrenosum associated with Crohn’s’s disease in a Malawian teenage boy: case report and review of literature. Trop Doc. 2018; 48: 43–46 10.1177/0049475517724972 28767000

[36] Lee JH Chang IK Lee HE. Treatment of recalcitrant pyoderma gangrenosum with ulcerative colitis by adalimumab injection. Ann Dermatol. 2017; 29: 260–262 28392668 10.5021/ad.2017.29.2.260 PMC5383766

[37] Diatta BA Toumbou FF Diop A. Pyoderma gangrenosum among children in Senegal: 6 cases. Our Dermatol Online. 2017; 8: 463–466

[38] Kechichian E Haber R Mourad N. Pediatric pyoderma gangrenosum: a systematic review and update. Internat J Dermatol. 2017; 56: 486–495 10.1111/ijd.13584 28233293

[39] Ormerod AD Thomas KS Craig FE. Comparison of the two most commonly used treatments for pyoderma gangrenosum: results of the STOP GAP randomised controlled trial. Br Med J. 2015; 350: h2958 26071094 10.1136/bmj.h2958 PMC4469977

[40] Weizman A Huang B Berel D. Clinical, serologic, and genetic factors associated with pyoderma gangrenosum and erythema nodosum in inflammatory bowel disease patients. Inflamm Bowel Dis. 2014; 20: 525–533 24487271 10.1097/01.MIB.0000442011.60285.68 PMC4046633

[41] Ellinghaus E Ellinghaus D Stuart PE. Genome-wide association study identifies a psoriasis susceptibility locus at TRAF3IP2. Nat Genet. 2010; 42: 991–995 20953188 10.1038/ng.689 PMC3136364

[42] Phillips FM Verstockt B Sebastian S. Inflammatory cutaneous lesions in inflammatory bowel disease treated with Vedolizumab or Ustekinumab: an ECCO CONFER multicentre case series. J Crohn’s Colitis. 2020 Apr 22] 10.1093/ecco-jcc/jjaa078 32318735

[43] Schwandner R Yamaguchi K Cao Z. Requirement of tumor necrosis factor receptor-associated factor (TRAF)6 in interleukin 17 signal transduction. J Exp Med. 2000; 191: 1233–240 10748240 10.1084/jem.191.7.1233 PMC2193168

[44] Ciccacci C Biamcone L Di Fusco D. TRAF3IP2 gene is associated with cutaneous extraintestinal manifestations in inflammatory bowel disease. J Crohn’s Colitis. 2013; 1: 44–52 10.1016/j.crohns.2012.02.020 22445837

[45] Nguyen TV Cowen EW Leslie KS. Autoinflammation: from monogenic syndromes to common skin diseases. J Am Acad Dermatol. 2013; 68: 834–853 23453357 10.1016/j.jaad.2012.11.002 PMC7512013

[46] Lee JI Park HJ Lee JY Cho BK. A case of pyoderma gangrenosum with ulcerative colitis treated with mesalazine. Ann Dermatol. 2010; 22: 422–425 21165212 10.5021/ad.2010.22.4.422 PMC2991719

[47] Regueiro M Valentine J Plevy S. Infliximab for treatment of pyoderma gangrenosum associated with inflammatory bowel disease. Amer J Gastroenterol. 2003; 98: 1821–826 12907338 10.1111/j.1572-0241.2003.07581.x

[48] Brooklyn T. Dunnill MGS. Shetty A. Infliximab for the treatment of pyoderma gagnrenosum: a randomized, double blind, placebo-controlled trial. Gut. 2006; 55: 505–509 16188920 10.1136/gut.2005.074815 PMC1856164

[49] Ikeda H Ishimaru Y Takayasu H. Efficacy of granulocyte apheresis in pediatric patients with ulcerative colitis: a pilot study. J Pediatr Gastroenterol Nutr. 2006; 43: 592–596 17130733 10.1097/01.mpg.0000237928.07729.79

[50] Rolandsdotter H Eberhardson M. Fagerberg U Finkel Y. Granulocyte and monocyte aphresis for induction of remission in children with new-onset inflammatory bowel colitis. J Pediatr Gastroenterol Nutr. 2018; 66: 84–89 28604509 10.1097/MPG.0000000000001641

[51] Plumptre I Knabel D Tomecki K. Pyoderma gangrenosum: a review for the gastroenterologist. Inflamm Bowel Dis. 2018; 24: 2510–517 29788368 10.1093/ibd/izy174

[52] Oka M. Pyoderma gangrenosum and interleukin-8. Br J Dermatol. 2007; 157: 1279–1281 17916207 10.1111/j.1365-2133.2007.08202.x

[53] Kim DH Cheon JH. Pathogenesis of inflammatory bowel disease and recent advances in biologic therapies. Immune Netw. 2017; 17: 25–40 28261018 10.4110/in.2017.17.1.25 PMC5334120

[54] Arivarasan K Bhardwaj V Sud S. Biologics for the treatment of pyoderma gangrenosum in ulcerative colitis. Intestinal Research. 2016; 14: 365–368 27799888 10.5217/ir.2016.14.4.365 PMC5083266

[55] Vavricka SR. Gubler M Gantenbein C. Anti-TNF treatment for extraintestinal manifestations of inflammatory bowel disease in the Swiss IBD cohort study. Inflamm Bowel Dis. 2017; 23: 1174–1181 28452862 10.1097/MIB.0000000000001109

[56] Chatzinasiou F Polymeros D Panagiotou M. Generalized pyoderma gangrenosum associated with ulcerative colitis: successful treatment with infliximab and azathioprine. Acta Dermatovenerol Croat. 2016; 24: 83–85 27149138

